# Antimicrobial Activity of the Peptide C14R Against Ab Initio Growing and Preformed Biofilms of *Candida albicans*, *Candida parapsilosis* and *Candidozyma auris*

**DOI:** 10.3390/biom15030322

**Published:** 2025-02-21

**Authors:** Jan-Christoph Walter, Ann-Kathrin Kissmann, Daniel Gruber, Daniel Alpízar-Pedraza, Ernesto M. Martell-Huguet, Nico Preising, Armando Rodriguez-Alfonso, Ludger Ständker, Christoph Kleber, Wolfgang Knoll, Steffen Stenger, Carolina Firacative, Frank Rosenau

**Affiliations:** 1 Institute of Pharmaceutical Biotechnology, Ulm University, 89081 Ulm, Germany; jan-christoph.walter@uni-ulm.de (J.-C.W.); ann-kathrin.kissmann@uni-ulm.de (A.-K.K.); daniel.gruber@uni-ulm.de (D.G.); dalpizarp@gmail.com (D.A.-P.); nestmartell@gmail.com (E.M.M.-H.); 2 Center for Pharmaceutical Research and Development (CIDEM), 26th Avenue, No. 1605, Nuevo Vedado, La Habana 10400, Cuba; 3 Core Facility for Functional Peptidomics (CFP), Faculty of Medicine, Ulm University, 89081 Ulm, Germany; nico.preising@uni-ulm.de (N.P.); armando.rodriguez-alfonso@uni-ulm.de (A.R.-A.); ludger.staendker@uni-ulm.de (L.S.); 4 Core Unit of Mass Spectrometry and Proteomics, Faculty of Medicine, Ulm University, 89081 Ulm, Germany; 5 Faculty of Medicine and Dentistry, Danube Private University, Steiner Landstraße 124, 3500 Krems an der Donau, Austria; christoph.kleber@dp-uni.ac.at (C.K.); wolfgang.knoll@dp-uni.ac.at (W.K.); 6 Institute for Medical Microbiology and Hygiene, University Hospital Ulm, 89081 Ulm, Germany; steffen.stenger@uniklinik-ulm.de; 7 Studies in Translational Microbiology and Emerging Diseases (MICROS) Research Group, School of Medicine and Health Sciences, Universidad de Rosario, Bogota 111221, Colombia; cfiracative@gmail.com

**Keywords:** novel antifungals, infectious disease, next-generation antimycotic

## Abstract

Biofilms are the predominant lifeforms of microorganisms, contributing to over 80% of infections, including those caused by *Candida* species like *C. albicans*, *C. parapsilosis* and *Candidozyma auris*. These species form biofilms on medical devices, making infections challenging to treat, especially with the rise in drug-resistant strains. *Candida* infections, particularly hospital-acquired ones, are a significant health threat due to their resistance to antifungals and the risk of developing systemic infections (i.e., sepsis). We have previously shown that C14R reduces the viability of *C. albicans* and *C. auris*, but not of *C. parapsilosis*. Here, we show that C14R not only inhibits viability by pore formation, shown in a resazurin reduction assay, and in a *C. parapsilosis* and fluorescence-based permeabilization assay, but it also halts biofilm maturation and significantly reduces the biomass of preformed biofilms by over 70%. These findings suggest C14R could be an effective option for treating severe fungal infections, offering a potential new treatment approach for biofilm-related diseases. Further research is needed to fully understand its biofilm dispersal potential and to optimize its use for future applications as an antifungal in clinical settings.

## 1. Introduction

**Figure 1:**
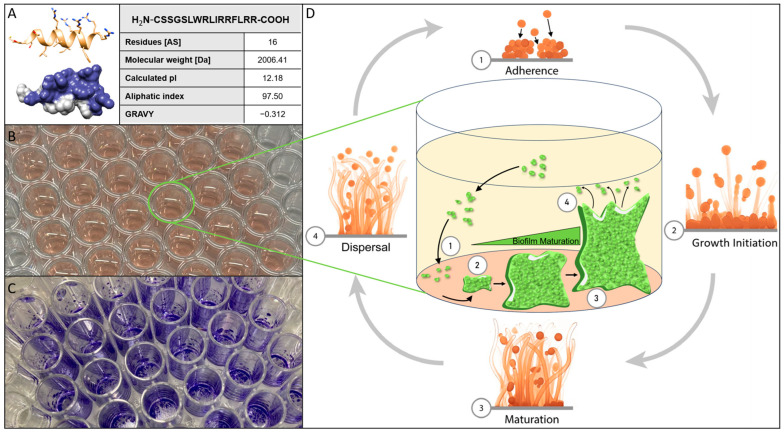
(**A**) Three-dimensional prediction gained via an ab initio method using AlphaFold2 from Google Colabs (https://colab.research.google.com/github/sokrypton/ColabFold/, 19 February 2025) of the peptide C14R with the sequence NH_2_-CSSGSLWRLIRRFLRR, as well as the following properties: amino acid sequence, length, theoretical isoelectric point (pI), the aliphatic index and the grand average hydropathy index (GRAVY) of C14R calculated with ExPASy ProtParam. (**B**) Example of a 96-well polystyrene microtiter plate (Sarstedt AG & Co. KG, Nümbrecht, Germany) filled with 200 µL of RPMI-1640 media and inoculated with cells of *C. albicans*. (**C**) Example of a 96-well microtiter plate (Sarstedt AG & Co. KG, Nümbrecht, Germany) after performing the staining of *C. albicans* biofilm cells with crystal violett. (**D**) Schematic overview of the lifecycle of *Candida* biofilm in four steps: adherence, growth initiation, maturation and dispersal.

Biofilms formed on abiotic and biotic surfaces are nowadays accepted as the lifeform of microorganisms of the highest relevance and are probably the “normal” way of living in general despite generations of microbiologists considering the traditional microbiological cultivation in Erlenmeyer flasks to be the most favorable condition for their research organisms [1,2,3,4,5]. In theory, this also holds true for all pertinent pathogenic bacteria and (fungal) eukaryotes. According to estimates, biofilms could be responsible for over 80% of all microbial infections and 65% of hospital-acquired infections [6,7,8]. The ability of microbial biofilms to significantly increase resistance to physical, chemical, and pharmacological stresses is a property that is almost universally acknowledged, making them a major threat in the clinical context, thereby posing considerable socio-economic challenges to health systems not only in underdeveloped countries [9,10]. The commensal yeast *Candida albicans* is supposedly the most prevalent and important species in clinical fungal infections, making fungi of this genus a class of extremely significant pathogens [11,12,13]. Up to 75% of women will face candidiasis at some point in their lives, and *C. albicans* is responsible for 85–95% of these infections in this cohort of patients [14]. Over the past few decades, other *Candida* species have become more prevalent in causing severe infections, but the dominant involvement of *C. albicans* in invasive infections appears to diminish [15,16,17,18,19]. *C. parapsilosis* is also of high significance among pathogenic yeasts as it can establish persistent biofilms on catheters, other implanted medical equipment, and generally on any biotic or abiotic surface in a medical facility, posing serious risks to patients, particularly upon invasive surgery. Furthermore, as *C. parapsilosis* is perfectly metabolically sustained by the complete parenteral nutrition of patients (e.g., in intensive care units), it represents a significant risk to undernourished infants and newborns with low birth weights [15,16,20,21,22]. Additionally, it has been documented that several clinical isolates of this species become less susceptible even to traditional types of antifungal medications, severely restricting the effectiveness of established treatment regimens [23,24,25,26]. However, *Candidozyma auris* [27], which was initially isolated from a Japanese patient in 2009, is a more recent pathogenic yeast and is, to date, still among the rarest types of clinically relevant *Candida* species [28]. In hospitalized patients, *C. auris* can result in serious bloodstream infections with a strikingly high mortality rate of 35% to 60% [29,30]. The fact that strains of *C. auris* with diverse drug resistances (likely due to the activation of ABC-type efflux pumps [31]) against common antifungals have independently emerged in various nations/continents across the world [32,33,34,35,36,37] poses a unique challenge to global pharmacology. As a result, *C. auris* has been identified as an emergent “superbug” by both the European Center for Disease Control (ECDC) and the U.S. Center for Disease Control (CDC), who have issued clinical advisories and started a widespread public conversation [38]. In 2022, the World Health Organization (WHO) published a new and alarming ranking of “fungal priority pathogens”, *C. albicans*, *C. auris*, and *C. parapsilosis* being at the top of this “hit list” [39]. ABC-type MFS (major facilitator superfamily) efflux pumps have been implicated in multidrug resistance of *C. auris* to traditional antifungal medications such as fluconazole or amphotericin B [31] and they are especially overexpressed in *C. auris* when it resides in biofilms [40]. These reports suggest that—in addition to resistance to traditional antifungal drugs—biofilm formation and the ensuing increase in physiological robustness are important aspects of virulence that help both common and uncommon *Candida* species to successfully establish their full pathogenic potential. The treatment of biofilm-based infections is particularly challenging as, currently, there are no medications that specifically target biofilms of *Candida* species or any other microorganism [41,42,43,44]. We believe that although systematic research to isolate dedicated anti-biofilm medication compounds against the *Candida* species is still underestimated in its importance by the scientific community, it is urgently needed. Essentially, the development of complex microbial biofilms can be divided into four stages: the attachment of planktonic cells to the biotic or abiotic biofilm substratum (adherence), cell assembly or aggregation and subsequent growth into structures resembling microcolonies (growth initiation), the production of species-specific mature biofilm architectures (maturation), and the release of the former biofilm cells into the planktonic phase (dispersal) (Figure 1). Antimicrobial peptides (AMPs) are a promising class of novel pharmaceutical compounds that can be used to combat both planktonic *Candida* cells and their biofilms [45,46,47,48,49,50]. The majority of AMPs, including the peptide C14R, reduce the functional integrity of microbial cell membranes by interacting with negatively charged phospholipids, thereby integrating into the lipid bilayer, forming pore-like structures and thus disintegrating the functional architecture of the cell envelope [51,52,53]. C14R is a derivative of the previously published antibacterial peptide BP100. It consists of an amphiphilic sequence of 16 amino acid residues with a molecular weight of 2 kDa and a predicted secondary structure of a single α-helix (Figure 1A) [51,53,54,55,56]. In our previous studies, C14R has proven its potential to reduce cell viability of the Gram-negative human pathogens *Pseudomonas aeruginosa* and *C. albicans,* as well as *C. auris*, including clinical isolates of these highly relevant species [51,53]. The principal activity of cell killing does not necessarily coincide with the ability to inhibit biofilm growth and/or maturation, especially not with biofilm dispersal. The latter process represents a decay of these elaborate structures by releasing viable cells into the environment, which can colonize new habitats, a physiological event with enormous relevance for virulence [57]. In this short communication, we want to share initial but, in our opinion, promising results demonstrating, for the first time, that C14R can inhibit the growth of *C. albicans*, *C. auris*, and *C. parapsilosis* biofilms. More important is the finding that not only ab initio growth of biofilms was inhibited in the presence of C14R, but the maturation of *C. albicans* biofilms could be arrested, and preformed biofilms of *C. auris* and *C. parapsilosis* could even be reduced in their biomass to less than 30%. We believe that C14R alone or in combination with other (peptide) drugs may be a valuable option for the development of new treatment regimens for these severe fungal infections.

## 2. Materials and Methods

### 2.1. Materials

Acetic acid, crystal violet, 3-(N-morpholino) propanesulfonic acid (MOPS), and paraformaldehyde were obtained from Carl Roth GmbH (Karlsruhe, Germany). The fluorescent dye fluorescein isothiocyanate (FITC) and RPMI-1640 medium containing L-glutamine were acquired from Thermo Fisher Scientific (Waltham, MA, USA). Resazurin sodium salt was procured from Sigma-Aldrich Chemie GmbH (Steinheim, Germany). Phosphate-buffered saline (PBS) was purchased from Life Technologies (Carlsbad, CA, USA). The peptide C14R was chemically synthesized by the Core Facility Functional Peptidomics (University of Ulm, Ulm, Germany).

### 2.2. Peptide Synthesis

C14R was synthesized as previously described in the work of Mildenberger et al., 2024 [57]. In brief, the protocol included automated synthesis on a solid phase, purification by HPLC and quality control by LC-MS and MALDI-TOF.

### 2.3. Candida Cultivation

For each experiment, the strain of interest of *C. albicans* (ATCC90028), *C. auris* (DSMZ-No. 21092) or *C. parapsilosis* (ATCC22019) was cultivated by inoculating 100 µL from a cryoprotectant glycerol culture in 5 mL RPMI-1640 media at 37 °C for 18 h and orbital shaking at 150 rpm.

### 2.4. Biofilm Kinetic Assay/Crystal-Violet-Assay

To analyze the kinetics of biofilm formation, 2.5 × 10^3^ cells of *C. albicans*, *C. auris* and *C. parapsilosis* were inoculated in 200 µL of RPMI-1640 medium in a flat-bottomed 96-well polystyrene microtiter plate (Sarstedt AG & Co. KG, Nümbrecht, Germany) and incubated at 37 °C without agitation. Biofilm formation was quantified at 2, 4, 6, 8, 24, 48, 72 and 96 h of incubation in triplicates using the crystal violet assay initially developed for bacterial biofilms by George O’Toole [58] and widely applied to *Candida* biofilms [59,60,61]. In short, the planktonic phase was removed and biofilms were washed twice with 200 µL of water. The biofilms were then stained for 15 min with 200 µL of a 0.1% (*w*/*v*) crystal violet solution. After removing the supernatant, the biofilms were washed twice with 200 µL of water. The stained biofilms were air-dried for 24 h at 25 °C and the remaining crystal violet was dissolved in 200 µL of 30% acetic acid for 15 min and transferred into a new 96-well plate. Subsequently, absorbance was measured at 560 nm using a Tecan Infinite F200 microplate reader (Tecan Group Ltd., Männedorf, Switzerland) to quantify the biofilm biomass. At the given time points, the planktonic phase, as well as the biofilms, were examined by phase-contrast microscopy at 400× magnification with a Leica DMi8 inverted fluorescent microscope (Leica Microsystems CMS GmbH, Wetzlar, Germany). Additionally, the OD_600_ value of the planktonic phase was measured at the time points 0, 2, 8, 24, 48, 72, and 96 h of incubation using a Tecan Infinite F200 microplate reader (Tecan Group Ltd., Männedorf, Switzerland).

### 2.5. Resazurin Reduction Assay/Viability Assay

The viability of *C. parapsilosis* cells in the presence of various concentrations of C14R (0–200 µg/mL), as well as Amphotericin B (2 µg/mL), was assessed similarly to the “Clinical and Laboratory Standards Institute” guidelines of the M27-A3 broth microdilution assay [62] by incubating 2.5 × 10^3^ yeast cells in triplicates in 200 µL of C14R-supplemented RPMI-1640 media in flat-bottomed 96-well polystyrene microtiter plates (Sarstedt AG & Co. KG, Nümbrecht, Germany) at 37 °C for 24 h and orbital shaking at 900 rpm on an Eppendorf shaker. Viability was quantified using the resazurin reduction assay [63,64]. In short, the cells were incubated for 2 h at 37 °C with 20 µL of a 0.15 mg/mL resazurin solution. Viable cells convert resazurin to resorufin, the fluorescence of which can be measured at an excitation wavelength of 535 nm and an emission of 595 nm with a Tecan infinite F200 microplate reader (Tecan Group Ltd., Männedorf, Switzerland). A sterility control was performed in triplicate and subtracted as a blank from the recorded values.

### 2.6. Permeabilization Assay

A permeabilization assay to assess the ability of C14R to form pores in the membranes of *C. auris* and *C. parapsilosis* was performed as described previously [51,53]. For this purpose, the fluorescent dye fluorescein isothiocyanate (FITC), with a molecular weight of 389 Da, was utilized. Then, 10^7^ cells of *C. auris* and *C. parapsilosis* were incubated in triplicates at 37 °C in 200 µL RPMI-1640 media supplemented with 12 µg/mL of C14R. Non-supplemented RPMI was used as a control. After 2 h of incubation, the cells were centrifuged at 11,000× *g*, and the resulting pellet was washed once with PBS. The cells were then incubated at room temperature with 200 µL of FITC at a final concentration of 125 µg/mL in 1× PBS for 20 min, followed by centrifugation at 11,000× *g* for 2 min. After discarding the supernatant, the yeast cells were fixed by incubation with 4% (*w*/*v*) paraformaldehyde in PBS for 10 min and washed three times with PBS. Eventually, the resuspended cells in 200 µL PBS were transferred to a flat-bottomed polystyrene 96-well microtiter plate (Sarstedt AG & Co. KG, Nümbrecht, Germany) and fluorescence was measured using a Tecan SPARK microplate reader (Tecan Group Ltd., Männedorf, Switzerland) at an excitation wavelength of 498 nm and emission wavelength of 517 nm.

### 2.7. Inhibition of Biofilm Formation Assay

The analysis of the peptides’ antifungal effect on biofilm formation was evaluated under similar conditions, as previously outlined [58,61,64]. In summary, triplicates of 2.5 × 10^3^ cells were incubated in 200 µL of RPMI-1640 media, supplemented with the corresponding peptide (0–200 µg/mL) and Amphotericin B (2 µg/mL) concentrations, in flat-bottomed 96-well polystyrene microtiter plates (Sarstedt AG & Co. KG, Nümbrecht, Germany) at 37 °C for 24 h. Afterward, the crystal violet assay was performed as previously described. A sterility control was subtracted as a blank from the recorded values.

### 2.8. Decay of Preformed Biofilm Assay

An experiment was conducted to assess the ability of C14R to decay preformed *Candida* biofilms. In the first phase, 2.5 × 10^3^ *Candida* cells were incubated in 200 µL of RPMI-1640 media in a flat-bottomed 96-well polystyrene microtiter plate (Sarstedt AG & Co. KG, Nümbrecht, Germany) at 37 °C for 24 h without agitation. The biomass of the formed biofilms was quantified using the crystal violet assay. Biofilm formation was quantified in triplicate for each strain of *Candida* and served as a reference for biofilm formation within 24 h.

In the second phase, the supernatants in the remaining wells of the first phase were carefully replaced with 200 µL of RPMI-1640 medium containing Amphotericin B (2 µg/mL) or C14R at concentrations ranging from 0 to 400 µg/mL (also in triplicates). These wells were then incubated at 37 °C for an additional 24 h to allow for C14R treatment. After this second incubation phase, the crystal violet assay was repeated to assess any reduction in the biofilm mass, comparing it to the reference biofilm mass before C14R treatment.

### 2.9. Statistical Analyses

All experiments were reproduced twice (*n* = 3). By a Student’s *t*-test (unpaired), the significance was determined in the statistics. In addition, 0.05 > *p* values were deemed significant. * = *p* < 0.05, ** = *p* < 0.01, *** = *p* < 0.001 and **** = *p* < 0.0001.

## 3. Results

**Figure 2:**
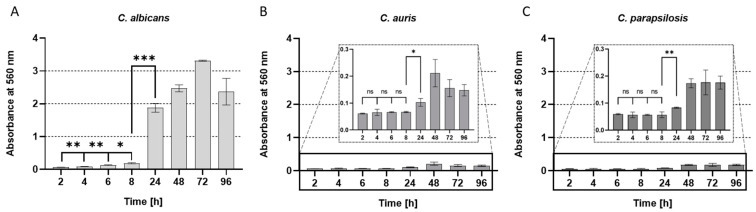
Kinetic of biofilm formation at the time points 2, 4, 6, 8, 24, 48, 72 and 96 h of (**A**) *C. albicans*, (**B**) *C. auris,* and (**C**) *C. parapsilosis*, all determined using the crystal violet assay. All experiments were conducted in triplicate with error bars representing standard deviations.. *p* values < 0.05 were considered significant. ns denotes not significant. * denotes *p* < 0.05, ** denotes *p* < 0.01, and *** denotes *p* < 0.001.

Formation of (elaborate) biofilms is the physiological process involving four distinct phases, adherence, growth initiation, maturation, and dispersal as the final phase, initiating the repetition of this process on new surfaces. The time needed for microbes to build up the final stages of the respective biofilm is individually different, and it depends on the species that have intrinsic abilities to form biofilms, the surface involved, the medium, and the incubation regime (i.e., aeration). The reference strains of *C. albicans*, *C. auris*, and *C. parapsilosis* were allowed to form biofilms on the polystyrene surfaces of commercial microtiter-plates in Roswell Park Memorial Institute (RPMI) 1640 Medium [62], which was originally developed for human cell culture techniques and thus represents a reasonable approximation of nutritional conditions the pathogenic yeasts will normally face in the human body (e.g., on freshly infected wounds). The kinetics of biofilm development and thus the increase in biomass as a measure for the fungal growth on the abiotic surface polystyrene were recorded for 96 h with sampling intervals of two hours in the adherence phase and 24 h in later stages of the biofilm formation process, as proven reasonable in our previous studies [64,65,66,67,68]. *C. auris* and *C. parapsilosis* formed clearly detectable biofilms at comparable intensities for each of these species (Figure 2B,C). However, the *C. auris* and *C. parapsilosis* biofilms produced drastically lower amounts as compared to *C. albicans* (Figure 2A), which is one of the most prominent biofilm-forming yeasts in the literature [69]. As expected, *C. albicans* showed the most pronounced biofilm formation capability under these conditions, with a kinetic following the scheme that a “lag-phase” occurred between inoculation (*t* = 0 h and 8 h), which, in this case, represented the adherence and early growth initiation phases at the onset of biofilm development, then proceeding into the maturation phase. This important switching point was accordingly observable after a growth period of 24 h, thereby qualifying the period 24–48 h as the most relevant and critical stage [57]. Accordingly, the cell numbers (measured as the optical density [O.D._600_] spectrophotometrically at a wavelength of 600 nm) in the liquid part of the cultures (i.e., the planktonic phase) were significantly higher in relation to the respective biofilm masses for *C. auris* and *C. parapsilosis*, but rather limited for the best biofilm performer, *C. albicans* (Figure S1). The optical inspection by classical light microscopy of samples from the planktonic phases at each time point of the growth curves, as well as for the biofilms, was in agreement with the quantitative measurements and supported these results adequately (Figure S2).

**Figure 3:**
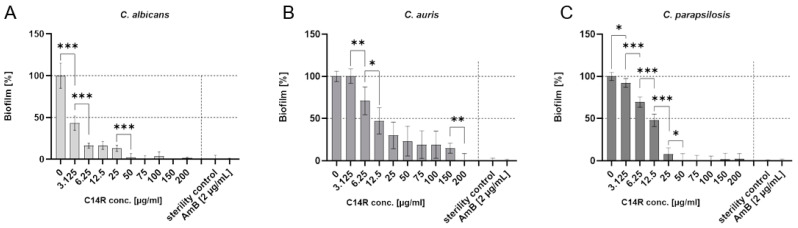
Inhibition of biofilm formation by incubation of *Candida* cells with the antimicrobial peptide C14R simultaneously with inoculation. Graphs show the formed biofilms after an incubation time of 24 h. Biofilm mass was analyzed using the crystal violet assay. The resulting effective MBIC dose was 50 µg/mL for *C. albicans* (**A**), 200 µg/mL for *C. auris* (**B**), and 50 µg/mL for *C. parapsilosis* (**C**). *p* values < 0.05 were considered as significant; * denotes *p* < 0.05; ** denotes *p* < 0.01; *** denotes *p* < 0.001 while ns denotes not significant.

The peptide C14R was shown to be functional as a classical pore-forming antimicrobial compound with the ability to kill pathogens like the bacterium *P. aeruginosa* and the yeasts *C. albicans* and *C. auris* [53]; however, this functionality of C14R for *C. parapsilosis* remained unaddressed and we decided to include this question in the study presented here. Therefore, we performed the respective measurement with cells grown in the planktonic phase also with this yeast and were able determine C14R to also be active against it with a peptide concentration of 175 µg/mL, sufficient to completely kill *C. parapsilosis* (Figure S3). The pore-forming ability in bacterial and yeast cells has previously been demonstrated using the cell (membrane) permeabilization assay using the otherwise not-membrane-penetrating fluorescent dye fluorescein isothiocyanate (FITC) [51,53]. Based on the previous experience with *C. albicans* [53], this permeabilization assay allowed for measurements of pore formation with the other strains and was also fully functional for planktonic *C. auris* and *C. parapsilosis,* and allowed us to verify C14R as a pore-forming antimicrobial peptide also for these yeasts (Figure S4). The ability to reduce the viability of planktonic cells does not necessarily allow for the conclusion that a respective compound also has anti-biofilm activities. This is especially the case if one intention is to support or induce the dispersal of preformed biofilms (i.e., destroy premature or matured elaborate architectures) with maturation times potentially playing an important role in this process. The principal ability to inhibit biofilm formation per se was shown by the growing biofilms of *C. albicans*, *C. auris* and *C. parapsilosis* completely in the presence of C14R, which was added simultaneously with the inoculation of the cultures, and the determination of biofilm mass using the crystal violet staining assay again. The MBIC (minimal biofilm inhibitory concentrations), i.e., the lowest concentration resulting in the complete inhibition of biofilm formation, was found to be 50 µg/mL of C14R for *C. albicans* and *C. parapsilosis* (Figure 3A,C), whereas *C. auris* required concentrations four times higher, of 200 µg/mL (Figure 3B). The total inhibition of biofilm growth in the presence of inhibitory compounds includes the very early phases of adherence to the respective surface, the subsequent establishment of productive cell–cell and/or cell–substratum interactions, which can be influenced by the respective drug.

**Figure 4:**
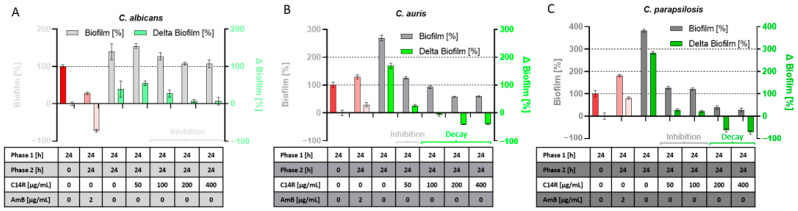
Inhibition of further biofilm growth in the incubation phase with C14R, phase 2 (24–48 h) compared to the biofilm mass of phase 1 (24 h of incubation without C14R, red bar). For *C. albicans* (**A**), an inhibition of gain in biofilm-biomass could be detected at concentrations ≥ 100 µg/mL. For *C. auris* (**B**), a concentration of 50 µg/mL led to an inhibition of further biofilm growth, while concentrations ≥ 100 µg/mL could decay the biomass compared to the phase 1 biofilm mass. The concentration for inhibition of further growth of *C. parapsilosis* (**C**) was 50–100 µg/mL, while ≥200 µg/mL led to a biofilm decay of *C. parapsilosis*.

In contrast, the undisturbed early development of biofilms towards the switching point (24–48 h) results in the onset of a maturation process leading to the final architecture and thus represents an essential phase providing stability and robustness to the cell community, which is only afterwards developed to perfection. Thus, growing biofilms in this phase (after 24 h) represent a challenging target for anti-biofilm compounds, which, in principle, can influence further growth or, in extreme cases, even induce biofilm decay or at least help to dissolve the preformed initial biofilm. To determine the effect of C14R on *C. albicans*, *C. auris*, and *C. parapsilosis* preformed biofilms, we divided the analysis into two distinct phases, with “phase 1” including the period from inoculation (*t* = 0 h) to 24 h without C14R (red bars in Figure 4), and “phase 2” including an additional 24 h (to 48 h finally) as a growth surplus in the presence of C14R (Figure 4A–C). The classical antifungal drug Amphotericin B served as a control since its mode of action, like for C14R, is also the disintegration of the fungal cell membrane. However, the activity of this polyene results from binding to ergosterol as an essential membrane phospholipid, indirectly leading to membrane instabilities and finally cell death. Although Amphotericin B was functional in reducing biofilm mass in phase 2 in cultures of *C. albicans*, it completely failed for *C. auris* and *C. parapsilosis* (Figure 4). In contrast, C14R was not able to reduce biofilm mass for *C. albicans*, but it managed to control and significantly inhibit further growth and intensifying biomass development in phase 2 in comparison to the untreated control, which, as expected, gained additional biofilm mass (Figure 4A). This effect was dose-dependent and appeared visible for concentrations ≥ 100 µg/mL. More important was the finding that C14R was not only active in inhibiting the gain of a surplus of *C. auris* and *C. parapsilosis* biofilm mass by further growth after the addition of the peptide, but was also shown to be functional in reducing biofilm mass in phase 2, which had been pre-produced in phase 1 at concentrations of ≥100 µg/mL for *C. auris* and ≥200 µg/mL for *C. parapsilosis* (Figure 4B,C, labeled with “decay”). These biofilm reductions in phase 2 were as drastic as 42% for *C. auris* and up to 73% for *C. parapsilosis* as compared to the respective untreated controls.

## 4. Discussion

Among clinically relevant *Candida* species, *C. albicans* undoubtedly remains the major pathogen and the most commonly isolated etiological agent of candidiasis [70,71]. However, its dominance is gradually being overtaken by non-albicans species, particularly *C. parapsilosis*, which exhibits a notable ability to form robust biofilms on both biotic and abiotic surfaces, such as medical devices like catheters and prostheses [15,16,22]. The WHO “alarmed” set of today’s high-priority fungal pathogens is completed by *C. auris,* which has had negative effects on human societies predominantly in tropic and subtropic regions, with the potential to spread into other regions of the world as the first multidrug-resistant fungal zoonosis emerging from climate change [72]. Consequently, it suits humanity well to redirect research to finding novel antimicrobial drugs dedicated not only to *Candida* species per se but also to the development of future biofilm-dedicated medications, also including the more-than-considerable virulence factor of forming biofilms reducing susceptibility towards antimicrobial agents and effective control of the pathogens by the immune system of the host.

Two different sets of experiments were performed to characterize the biofilm control activity of C14R on the three model *Candida* species. One set of experiments was performed to quantify biofilm growth and development when the peptide was present in the cultures from the beginning (added at inoculation). In the second set, biofilms were allowed to preform for one day, with the subsequent addition of the drug and measurement of further growth for one additional day. Inhibition of ab initio growing biofilms was effectively possible for the three species, with C14R concentrations leading to a complete decrease in biofilm mass after 24 h of 50 µg/mL for *C. albicans* and *C. parapsilosis* and 200 µg/mL for *C. auris*. Growing in biofilms provides (pathogenic) microbes with an increase in resistance towards antimicrobial compounds, which can be 10–1000-fold as compared to their planktonically growing counterparts, a fact that perfectly describes the challenge in clinical microbiology to fight those microbial-encapsulated communities (reviewed in [73]). The minimal inhibitory concentrations (MIC) of C14R have been determined in previous studies for sets of clinical isolates of *C. albicans* and *C. auris* in the range of 5 µg/mL [53], qualifying the biofilm inhibitory concentrations observed here (50–200 µg/mL) as perfectly convincing with respect to this general description. A challenging task widely accepted as such in the literature is the eradication of preformed biofilms as the next level of biofilm inhibition and control. In this regard, we performed assays for the inhibition of further growth and the contingent (and desired) induction of biofilm decay. C14R can prevent further growth of the preformed biofilms of *C. albicans* with high efficiency at concentrations above 100 µg/mL, as indicated by a remarkable reduction in detectable biomass. The preventive effect of C14R on biofilms gaining a surplus of mass was also present for *C. auris* and *C. parapsilosis* at concentrations above 50 µg/mL. However, the crucial difference became obvious at higher concentrations of the peptide when growth inhibition switched to a real reduction (i.e., a net decay) of the initial biofilm mass at the beginning of the experiment measured prior to the addition of C14R. Interestingly, Amphotericin B, as a non-peptide antifungal, which served as a control in these experiments, was able to reduce biofilms significantly in the case of *C. albicans* but failed completely in biofilm decay for *C. auris* and *C. parapsilosis*. This advantage of the polyene macrocyclic lactone Amphotericin B as a smaller and less charged molecule over C14R solely in the case of *C. albicans* may be primarily the result of an impaired penetration of antifungal agents within the biofilm structure, which has been discussed as a general property of microbial biofilms [74] with enhanced levels of extracellular chemical compounds (i.e., biopolymers) in the biofilm matrix, also designated the “matrixome” and functional as a molecular sieve [75]. Surprisingly, in contrast to the results for *C. albicans,* Amphotericin B was not functional to abolish biofilm on-growth and maturation for the biofilms of *C. auris* and *C. parapsilosis* with lower mass values. In-depth characterization of the C14R biofilm dispersal potential is required, including kinetic studies of the dose-dependent induction of biofilm decay and the release of cells including clinical isolates. A limitation of this study thus lies in the fact that the strains used were non-resistant laboratory reference strains. Regardless of the fact that the results presented here originate from experiments with to date, not optimized parameters, and taking into account that follow-up studies are required using large ensembles of clinical (and multi-drug resistant) isolates, measuring detailed dose dependencies and biofilm decay kinetics, the undoubtedly observed destruction of preformed biofilms qualifies C14R as a probable upcoming drug for the control of biofilms formed by *C. auris* and *C. parapsilosis*. Previously, we have demonstrated that a group of novel neutralizing peptides can effectively inhibit the formation of *C. albicans* biofilms [64,65,67,76]. We believe that C14R action can be further improved by molecular modifications and by combining it with these neutralizing peptides. This may open new avenues to eradicate *C. albicans* from biofilms with high efficiency, including multi-resistant variants of the common and highly pathogenic yeast.

## 5. Conclusions

With *C. parapsilosis,* another medically relevant species of *Candida* has been added to the target organisms for the peptide C14R, which was previously found to kill the two other species through its pore-forming ability. Also, for *C. parapsilosis,* the resazurin-based viability assay and the permeabilization assay showed a reduction in viability. Moreover, biofilm formation was inhibited by C14R for all species tested in this study with the, to date, unprecedented criterion that this effect was not limited to ab initio growing biofilms, being observed in preformed biofilms as well, the further growth of which was inhibited and these were even reduced, suggesting the induction of biofilm decay by C14R.

## Data Availability

The original contributions presented in this study are included in the article/Supplementary Materials. Further inquiries can be directed to the corresponding author.

## Supplementary Materials

The following supporting information can be downloaded at: https://www.mdpi.com/article/10.3390/biom15030322/s1, Figure S1: Kinetic of planktonic phase, measured OD_600_ values of the time points 0, 2, 8, 24, 48, 72 and 96 h are shown of (A) *C. albicans*, (B) *C. auris* and (C) *C. parapsilosis*. All experiments were conducted in triplicate with error bars representing standard deviations; Figure S2: Phase-contrast microscopy of the planktonic phase as well as the biofilms of (**A**) *C. albicans,* (**B**) *C. auris* and (**C**) *C. parapsilosis* at the time points 2, 4, 6, 8, 24, 48, 72 and 96 h and at 200× magnification. Scale bars indicate 50 µm.; Figure S3: Viability assay by incubation of *C. parapsilosis* cells with the antimicrobial peptide C14R simultaneously to inoculation. Graph shows the number of viable cells in percent compared to an untreated control after a total incubation time of 24 h. Viable cells were analyzed using the resazurin assay; Figure S4: Permeabilization assay of cells of *C. auris* and *C. parapsilosis*. Graph shows the fluorescence of *Candida* cells after the uptake of the fluorescent dye Fluorescein isothiocyanate (FITC). Fluorescence was sufficiently higher after an incubation time of 2 h with C14R compared to an untreated control. *p* values < 0.05 were considered as significant; ** denotes *p* < 0.01; *** denotes *p* < 0.001 while ns denotes not significant.
