# Supplementary material for: Antimicrobial Activity of the Peptide C14R Against Ab Initio Growing and Preformed Biofilms of Candida albicans, Candida parapsilosis and Candidozyma auris
Source: Biomolecules. 2025 Feb 21;15(3):322. doi: 10.3390/biom15030322 (PMC11939920; doi:10.3390/biom15030322)
Supplement: Supplementary file 1 [file biomolecules-15-00322-s001.zip › biomolecules-3441100-supplementary.pdf]

biomolecules

MDPI

Supplementary Materials

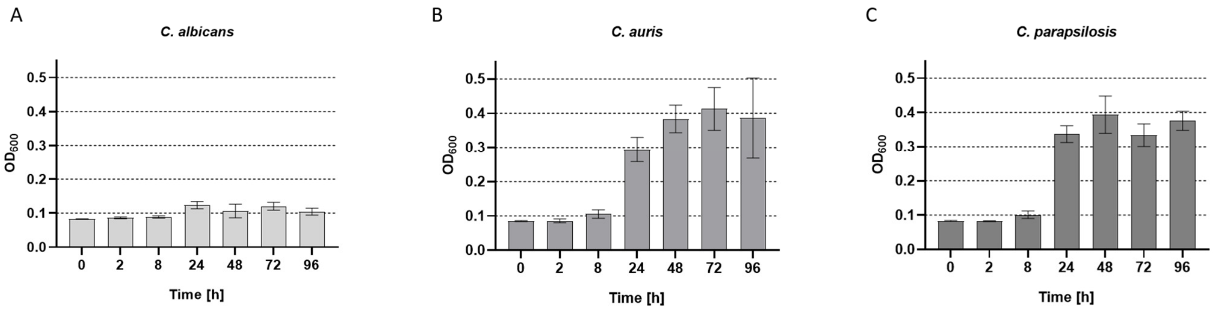

Figure S1. Kinetic of planktonic phase, measured OD_{600} values of the time points 0, 2, 8, 24,48,72 and 96 hours are shown of (**A**) *C. albicans*, (**B**) *C. auris* and (**C**) *C. parapsilosis*.

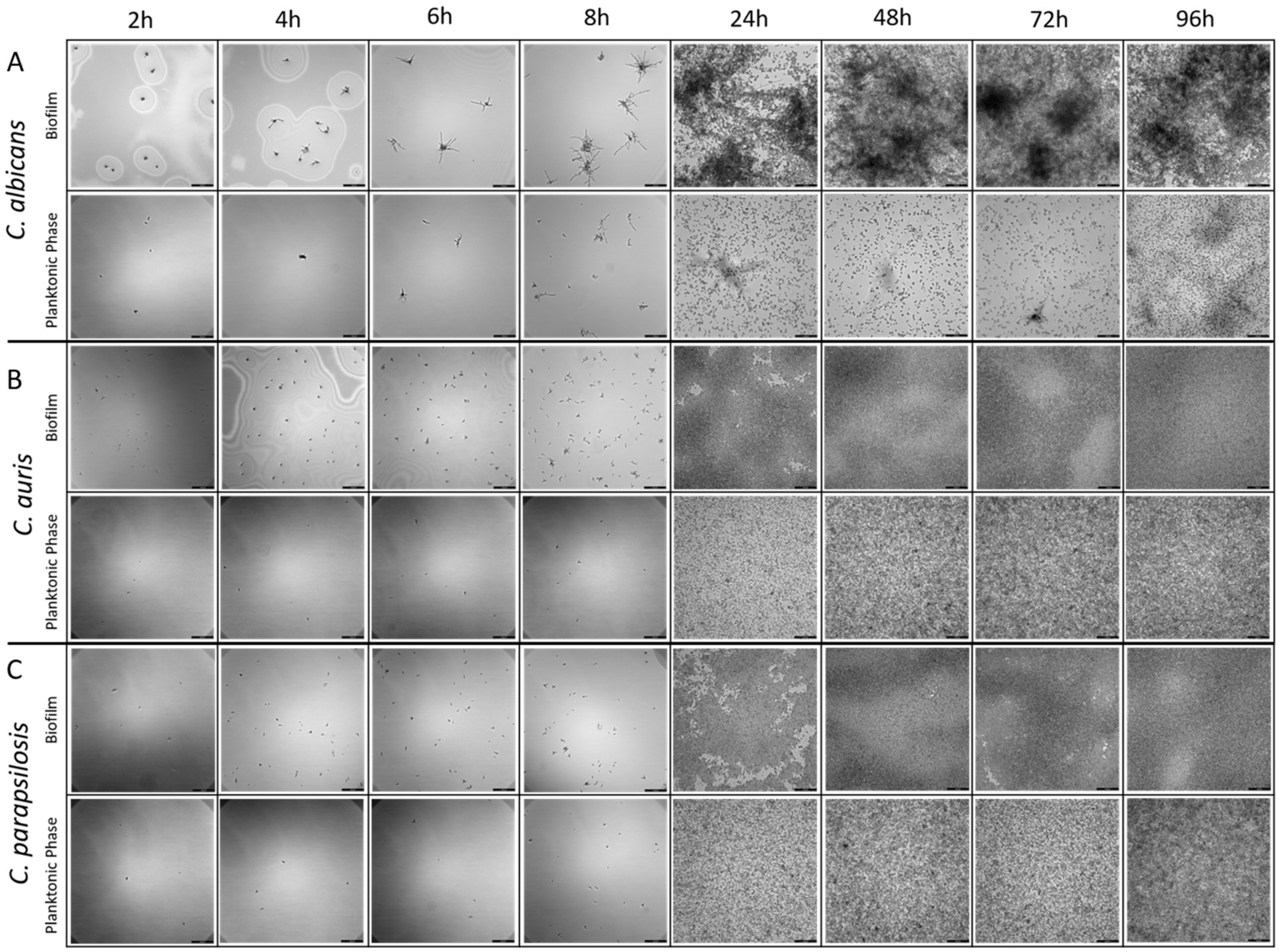

Figure S2. Phase-contrast microscopy of the planktonic phase as well as the biofilms of (**A**) *C. albicans*, (**B**) *C. auris* and (**C**) *C. parapsilosis* at the time points 2,4,6,8,24,48,72 and 96 hours and at 200× magnification.

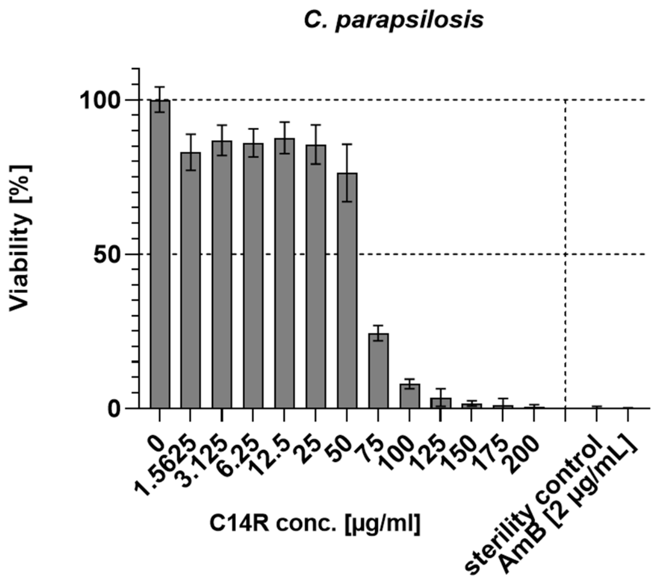

Figure S3. Viability assay by incubation of *C. parapsilosis* cells with the antimicrobial peptide C14R simultaneously to inoculation. Graph shows the number of viable cells in percent compared an untreated control after a total incubation time of 24 hours. Viable cells were analyzed using the resazurin assay.

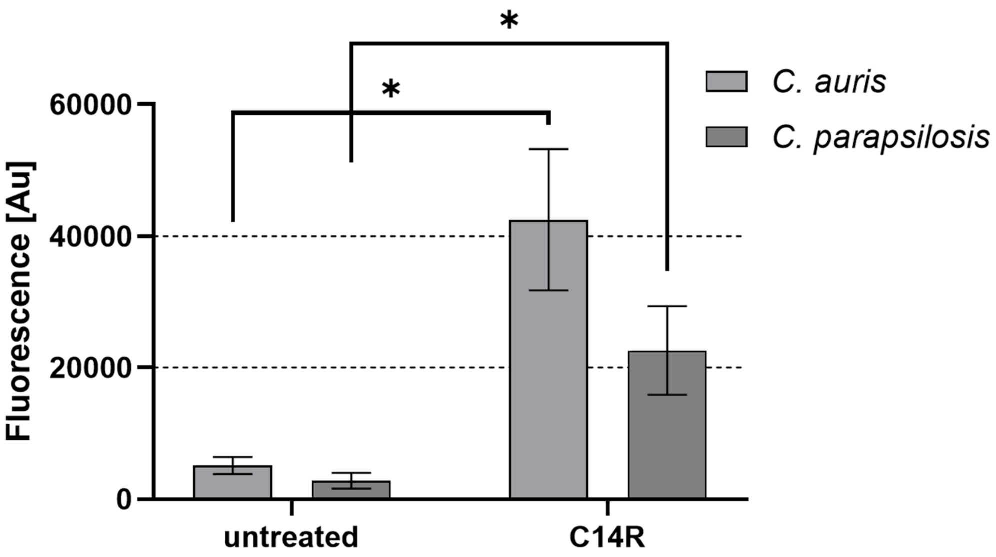

Figure S4. Permeabilization assay of cells of *C. auris* and *C. parapsilosis*. Graph shows the fluorescence of *Candida* cells after the uptake of the fluorescent dye Fluorescein isothiocyanate (FITC). Fluorescense was sufficiently higher after an incubation time of 2 h with C14R compared to an untreated control. P values < 0.05 were considered as significant; ** denotes p < 0.01; *** denotes p < 0.001 while ns denotes not siginificant
